# Is Skeletal Muscle Dysfunction a Limiting Factor of Exercise Functional Capacity in Patients with Sickle Cell Disease?

**DOI:** 10.3390/jcm10112250

**Published:** 2021-05-22

**Authors:** Etienne Gouraud, Philippe Connes, Alexandra Gauthier-Vasserot, Camille Faes, Salima Merazga, Solène Poutrel, Céline Renoux, Camille Boisson, Philippe Joly, Yves Bertrand, Arnaud Hot, Giovanna Cannas, Christophe Hautier

**Affiliations:** 1 Inter-University Laboratory of Human Movement Sciences (LIBM) EA7424, Team “Vascular Biology and Red Blood Cell”, University Claude Bernard Lyon 1, 69100 Villeurbanne, France; philippe.connes@univ-lyon1.fr (P.C.); alexandra.gauthier@ihope.fr (A.G.-V.); camille.faes@univ-lyon1.fr (C.F.); celine.renoux@chu-lyon.fr (C.R.); camille.boisson2@gmail.com (C.B.); philippe.joly@chu-lyon.fr (P.J.); giovanna.cannas@chu-lyon.fr (G.C.); christophe.hautier@univ-lyon1.fr (C.H.); 2 Laboratory of Excellence “GR-Ex”, 75015 Paris, France; 3 Institute of Universities of France, CEDEX 05, 75231 Paris, France; 4 Hematology and Oncology Pediatric Unit, University Hospital of Lyon, 69008 Lyon, France; yves.bertrand@ihope.fr; 5 Reference Centre in Sickle Cell Disease, Thalassemia and Rare Red Blood Cell and Erythropoiesis Diseases, Hospices Civils de Lyon, 69003 Lyon, France; salima.merazga@chu-lyon.fr (S.M.); solene.poutrel@chu-lyon.fr (S.P.); arnaud.hot@chu-lyon.fr (A.H.); 6 Internal Medicine Department, Edouard-Herriot Hospital, 69003 Lyon, France; 7 Laboratory of Biochemistry of Erythrocyte Pathologies, Biology Centre East, 69500 Bron, France

**Keywords:** skeletal muscle fatigue, electromyography, functional capacity, hemoglobin disorder

## Abstract

Patients with sickle cell disease (SCD) have reduced functional capacity due to anemia and cardio–respiratory abnormalities. Recent studies also suggest the presence of muscle dysfunction. However, the interaction between exercise capacity and muscle function is currently unknown in SCD. The aim of this study was to explore how muscle dysfunction may explain the reduced functional capacity. Nineteen African healthy subjects (AA), and 24 sickle cell anemia (SS) and 18 sickle cell hemoglobin C (SC) patients were recruited. Maximal isometric torque (Tmax) was measured before and after a self-paced 6-min walk test (6-MWT). Electromyographic activity of the *Vastus Lateralis* was recorded. The 6-MWT distance was reduced in SS (*p* < 0.05) and SC (*p* < 0.01) patients compared to AA subjects. However, Tmax and root mean square value were not modified by the 6-MWT, showing no skeletal muscle fatigue in all groups. In a multiple linear regression model, genotype, step frequency and hematocrit were independent predictors of the 6-MWT distance in SCD patients. Our results suggest that the 6-MWT performance might be primarily explained by anemia and the self-paced step frequency in SCD patients attempting to limit metabolic cost and fatigue, which could explain the absence of muscle fatigue.

## 1. Introduction

Sickle cell disease (SCD) is a group of genetic disorders characterized by the presence of at least one hemoglobin S (HbS) allele (p.Glu7Val in the hemoglobin β globin-subunit) and a second β globin-subunit pathogenic variant, resulting in pathological hemoglobin polymerization [1]. The most prevalent form of SCD is the homozygous sickle cell anemia (SS), where patients inherit two copies of HbS [2]. Under deoxygenated conditions, HbS is able to polymerize, creating rigid fibers that modify the morphology of the red blood cells (RBCs) into a crescent-like shape (i.e., sickling) [3]. Association of the HbS allele with the hemoglobin C (HbC) allele (p.Glu7Lys in β globin-subunit) leads to sickle cell-hemoglobin C disease (SC) [4]. In SC patients, HbC promotes RBCs dehydration through the activation of KCl transporter [5] and, as a result, facilitates HbS polymerization [6]. These sickled RBCs are very fragile and rigid, which may lead to chronic hemolytic anemia and frequent painful vaso-occlusive crises [3]. The two SCD genotypes are characterized by severe multiorganic complications leading to a reduced life expectancy. However, anemia is a milder, the frequency of vaso-occlusive-like complication is lower, and life expectancy is usually higher in SC compared to SS patients [7,8].

SCD patients display low functional capacity as demonstrated by the reduced distance performed by patients during a 6-min walk test (6-MWT) [9,10,11,12,13,14]. The 6-MWT is a submaximal exercise test used in a clinical setting to assess functional capacity in various chronic diseases [15,16]. In the past decade, several studies showed that the 6-MWT performance in SCD patients was influenced by the pulmonary capacity [17,18], anemia level [17,19,20,21], tricuspid regurgitation velocity [9,10,21,22,23] and the extent of hemorheological alterations [18,20]. Recent studies showed profound histological and functional alterations of the skeletal muscle in SCD patients such as amyotrophy, a decrease in oxidative capacity, a profound microcirculatory remodeling [24] and a reduction in muscle microcirculatory oxygenation [25,26]. Although both skeletal muscle strength and fatigability are strongly associated with the 6-MWT performance and functional capacity in various chronic diseases [27,28,29,30,31,32], the role of skeletal muscle in the functional capacity of SCD individuals has been poorly investigated and it is unknown whether the skeletal muscle is impacted by a submaximal exercise in this disease.

The aim of this study was to study the effects of a 6-MWT on the skeletal muscle function of healthy (AA), SS and SC individuals. The second aim was to explore the relationship between the 6-MWT distance and the skeletal muscle function. We hypothesized that SCD patients should display increased muscle fatigue during the 6-MWT, which could partly explain the reduced functional capacity.

## 2. Materials and Methods

### 2.1. Study Design and Patients

Nineteen African AA subjects, 24 SS and 18 SC patients were recruited to participate in this study. SCD patients were screened among patients in a clinical steady state (i.e., no acute vaso-occlusive crises, acute chest syndrome or hospitalization within the past 2 months, and no blood transfusion within the past 3 months) to verify the absence of exclusion criteria. They were followed at the University Hospital of Lyon (Hospices Civils de Lyon, Lyon, France), either by the Internal Medicine department (adults) or by the Haematology and Oncology Paediatric unit (adolescents). Exclusion criteria included: positive history of stroke or cerebral vasculopathy, leg ulcers, osteonecrosis of the femoral head, current pregnancy and pulmonary hypertension, shown by elevated tricuspid regurgitant jet velocity on doppler echocardiography. Physicians then proposed to eligible patients to participate to the study. The study was approved by the French Ethics Committee (CPP Est IV, Strasbourg, France, Clinical trial number: NCT03243812), and all experiments were performed according to the guidelines set by the Declaration of Helsinki. All subjects were volunteers and signed written informed consent (with parents for patients under 18 years old).

### 2.2. Experimental Design

Venous blood was drawn into EDTA tubes to determine hematological parameters. Transcutaneous oxygen saturation (SpO_2_), heart rate (HR) and blood pressure (BP) were measured at rest. Since SCD patients would be characterized by low physical activity level [33,34] (which could contribute to skeletal muscle deconditioning [35]), the physical activity level of the subjects was quantified with the Global Physical Activity Questionnaire (GPAQ) [36]. After a standardized warm-up, absolute maximal isometric torque (Tmax) of the knee extensors was measured at rest. Following a recovery period, they performed a 6-MWT and immediately after, Tmax was measured again to evaluate skeletal muscle fatigue.

### 2.3. Force Measurement

All participants performed a warm-up consisting of two bouts of 10 full knee extensions with weights of 2 and 4 kg, respectively, separated by a 2-min resting period. After this warm-up, they performed three isometric maximal voluntary contractions (iMVC) to determine Tmax of the quadriceps of the dominant leg, separated by a 2-min recovery to avoid any skeletal muscle fatigue [37]. They were asked to “push as hard and as fast as possible”, and strong verbal encouragement was provided to ensure production of their maximal torque. Tmax was measured with a strength dynamometer (DFS II, Chatillon Force Measurement, AMETEK STC, Elancourt, France) at a 90° knee joint and hip joint angle. In order to compare Tmax between groups, the highest value of Tmax measured in each subject throughout the study was selected.

### 2.4. 6-Minute Walk Test (6-MWT)

The self-paced 6-MWT was conducted according to the guidelines set by the American Thoracic Society [38]. Briefly, participants were instructed to perform the greatest distance in 6 min, i.e., walking as fast as they can without running. They walked between two marker cones situated 21 m apart. Standardized verbal encouragements were given every minute. HR, SpO_2_ and dyspnea, with the CR-10 Borg scale [39], were also determined before and immediately after the 6-MWT. The 6-MWT is particularly relevant since it has been widely used to determine functional status of SCD patients [9,10,13,20,40] and it reflects daily-life activities [15]. Predicted distance was calculated based on the equation of Burr et al. [41] and Geiger et al. [42] for adults and adolescents, respectively.

### 2.5. Electromyography

Electromyographic activity (EMG) of the *Vastus Lateralis* (VL) was recorded using surface electrodes (EMG Triode, nickel-plated brass, electrode diameter = 1 cm, inter-electrode distance = 2 cm, Thought Technology, Montreal, QC, Canada). EMG signal was sampled at 2048 Hz using the Flexcomp Infiniti system (Thought Technology, Montreal, QC, Canada). Before placing the electrodes, the skin was shaved, if needed, and cleaned with alcohol to improve the contact between the skin and the electrode and reduce skin impedance. Electrodes were placed on the VL belly according to the SENIAM guidelines [43]. Before analysis, raw EMG signals were filtered (Butterworth order 2, band pass from 10 to 500 Hz) and amplified with a gain of 500. Mean root mean squared (RMS) values were calculated with a 125 ms sliding window (Origin 2017, OriginLab, Northampton, MA, USA). Maximal mean RMS value (RMSmax) was measured during the plateau phase of the signal during iMVC. Each skeletal muscle contraction was detected during the walking test thanks to a threshold set at 10% of maximal RMS value, determining the onset and offset of the contraction. Then, mean RMS value was calculated and normalized by RMSmax. Step frequency of each subject was calculated as the number of EMG bursts detected per unit of time multiplied by 2 to take into account the fact that only one leg has been measured.

### 2.6. Statistical Analysis

All data were expressed as mean ± SD. The normality was checked with the D’Agostino–Pearson’s test. Comparison of data among the three groups was performed by a one-way ANOVA, followed by Bonferroni post hoc test or Kruskal–Wallis test, followed by Dunn post hoc test, depending on the normality of data. The effects of the 6-MWT on physiological variables were investigated and compared among the 3 groups by using a two-way ANOVA with repeated measurements followed by Bonferroni multiple comparisons. Spearman correlation was performed to test the association between several parameters. Finally, we performed a multiple linear regression model with all variables at *p* < 0.20 to identify independent predictors associated with the 6-MWT distance. The significance level was set at *p* < 0.05. Statistical analyses were performed using GraphPad Prism 8 (GraphPad Software, La Jolla, CA, USA).

## 3. Results

Anthropometrics, physiological, and hematological characteristics of the three groups are summarized in the Table 1. Diastolic blood pressure was significantly lower in both SS and SC compared to AA (*p* < 0.001 and *p* < 0.05, respectively). Leucocytes count was significantly higher in SS compared to AA (*p* < 0.05). Daily physical activity was not significantly different among the three groups. Both hematocrit and hemoglobin values were significantly different among the three groups: AA > SC > SS.

**Figure 1:**
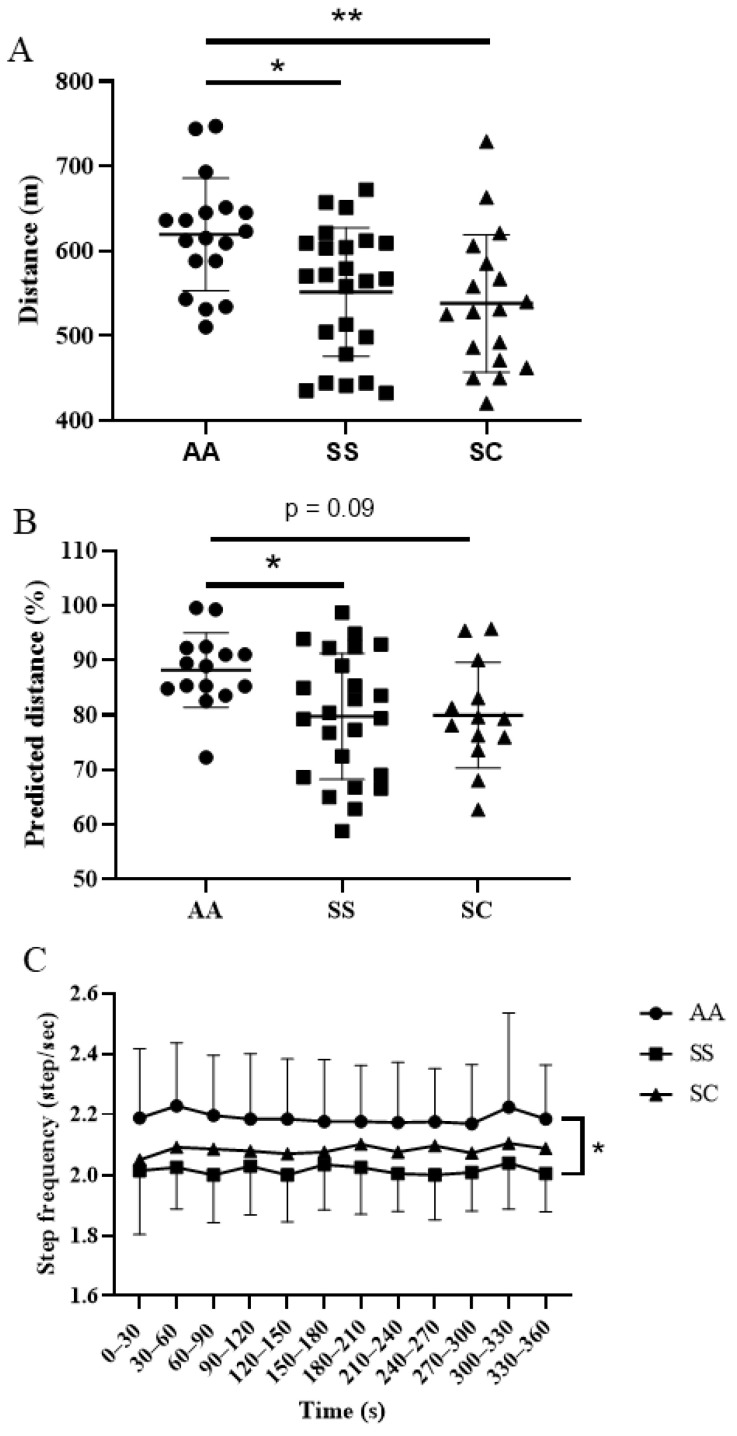
Absolute (**A**) and predicted (**B**) distance performed during the 6-MWT. Step frequency (**C**) for each group throughout the 6-MWT. Data are represented by Mean ± SD. *: *p* < 0.05; **: *p* < 0.01. 6-MWT: 6-min walk test.

The absolute distance walked during the 6-MWT was significantly lower in SS (*p* < 0.05) and SC (*p* < 0.01) patients (Figure 1A) compared to AA subjects but, when expressed in percentage of the predicted distance, the distance was lower in SS patients only (*p <* 0.05) and tended to be reduced in SC patients (*p* = 0.09) (Figure 1B). Interestingly, we found a genotype effect on step frequency with significantly lower values in SS patients compared to AA subjects throughout the test (*p* < 0.05) (Figure 1C) but, for each group, there were no modifications of step frequency within the 6-MWT.

**Figure 2:**
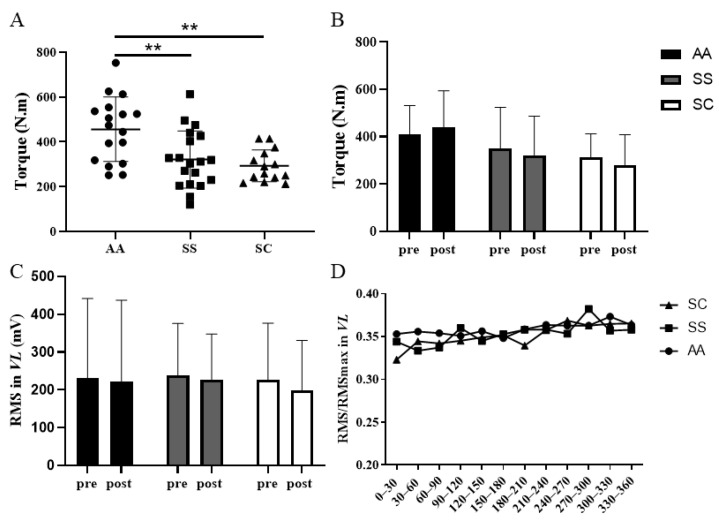
Absolute (**A**) maximal isometric torque at rest. Absolute maximal isometric torque (**B**) and RMS (**C**) values before and after the 6-MWT. RMS/RMSmax ratio of the *Vastus Lateralis* (**D**) during the 6-MWT for all groups. Data are represented by Mean ± SD. **: *p* < 0.01. RMS: Root mean square value.

At rest, Tmax0 of the knee extensors was significantly reduced in SCD patients (*p* < 0.01) (Figure 2A), but the 6-MWT did not induce any reduction in Tmax in the three groups (Figure 2B). Interestingly, there was a slight increase in Tmax in AA subjects (+5.27 ± 29.26%) while Tmax decreased in SS (−9.75 ± 12.72%, *p* < 0.05 vs. AA) and SC (−11.50 ± 19.06%, *p* < 0.05 vs. AA) patients. The RMS values were not different before and after the 6-MWT in the three groups (Figure 2C). The RMS/RMSmax ratio did not change significantly during 6-MWT and was not different among the three groups (Figure 2D). HR, SpO_2_ and dyspnea increased significantly following the 6-MWT in all groups but, at the end of the test, we observed no differences among the three groups.

In order to study the determinants of the 6-MWT performance, we first performed univariate analyses (i.e., Spearman correlation tests) in all SCD patients and in SS and SC patients separately. When pooled together, we found no correlation between the 6-MWT performance and Tmax (r = 0.26, *p* = 0.09), the percentage of Tmax loss (r = 0.13, *p* = 0.45), physical activity (r = 0.21, *p* = 0.22), hematocrit (r = 0.25, *p* = 0.11) and hemoglobin concentration (r = 0.16, *p* = 0.33). However, step frequency was strongly correlated with 6-MWT distance (r = 0.58, *p* < 0.001) in SCD patients. Then, we used a multilinear regression model to test if genotype, step frequency, Tmax and/or hematocrit were independent predictors of the 6-MWT distance. The model was highly significant (R² = 0.62, *p* < 0.0001) and genotype (*p* < 0.05), step frequency (*p* < 0.0001) and hematocrit (*p* < 0.05) were independent predictors of the 6-MWT distance. In each SCD genotype, univariate analysis showed that step frequency was positively correlated with the 6-MWT distance in SS (r = 0.62, *p* < 0.05) and SC patients (r = 0.56, *p* < 0.05). Interestingly, Tmax was moderately correlated with the 6-MWT distance in SS (r = 0.52, *p* < 0.05) but not in SC (r = −0.04, *p* = 0.87), while hematocrit was positively correlated with the 6-MWT distance in SC (r = 0.60, *p* < 0.01) but not in SS (r = 0.38, *p* = 0.06). Multivariate analysis showed that only step frequency (*p* < 0.05) remained an independent predictor of the 6-MWT in each genotype separately. No correlation between the 6-MWT distance and the different parameters was found in AA subjects.

## 4. Discussion

The main findings of this study were that (1) the 6-MWT did not induce skeletal muscle fatigue in SCD patients and (2) step frequency, genotype and hematocrit were independently associated with the 6-MWT test distance in SCD patients.

The absolute distance performed during the 6-MWT was significantly reduced in SCD patients as previously described [9,10,11,12,13,14], and we hypothesized that the apparition of skeletal muscle fatigue during the test could have participated in the lower distance reached by SCD patients. However, the 6-MWT did not result in skeletal muscle fatigue as shown by the absence of significant reduction in Tmax and/or modification in the RMS value during and after the 6-MWT. These results could be explained by the relatively low intensity of exercise that did not result in enough physiological perturbations to cause skeletal muscle fatigue. Interestingly, even though we observed no significant reduction in Tmax after the 6-MWT within each group, the percentage of change of Tmax was positive in AA subjects, while it was negative in both SS and SC patients. In SCD patients, it seems that the 6-MWT started to induce skeletal muscle fatigue, but the duration may be too short to induce enough perturbations to result in a significant loss in Tmax. Consequently, it can be hypothesized that walking at a higher speed or during longer duration could result in skeletal muscle fatigue in SCD patients.

During the past decades, several studies showed that the distance performed during the 6-MWT was modulated by pulmonary capacity [17,18], anemia [17,19,20], tricuspid regurgitation velocity [9,10,22,23] and the degree of hemorheological modifications [18,20]. In our study, we found that step frequency was an independent predictor of 6-MWT distance in SCD patients. These results are explained by the strong relationship between step frequency and walking speed [44,45] and as a consequence, higher step frequency results in a higher walking speed and distance performed during 6-MWT. It was previously shown in healthy subjects that step frequency and walking speed are chosen in order to minimize the metabolic cost of walking [46,47,48]. In pathological conditions and ageing, alterations in respiratory, cardiovascular and skeletal muscle systems result in higher energetic cost to perform daily life activities, causing higher sensations of fatigue [49,50,51]. Indeed, greater perceived fatigability is positively correlated with O_2_ cost of walking and negatively correlated with the 6-MWT distance in older women [52]. In our study, we observed lower step frequency in SS patients compared to healthy subjects; therefore, it could be hypothesized that SS patients chose a lower step frequency to limit the rise in metabolic cost and perceived fatigue during the 6-MWT. It could also explain the absence of skeletal muscle fatigue following 6-MWT in SS patients as they chose a step frequency to limit exercise-induced perturbations associated with skeletal muscle fatigue. However, our current results do not allow us to confirm this hypothesis, and further studies are needed to answer this question. We also observed that hematocrit level was an independent predictor of the 6-MWT as previously described in SS children and adults [17,20,21], and it was also shown that anemia is a major factor in the reduction of exercise capacity in SS patients [53,54]. During exercise, the capacity of the cardiovascular system to deliver O_2_ to the skeletal muscle is critical, and in SCD patients, anemia may reduce O_2_ delivery and limit exercise performance. Indeed, it was previously shown that blood transfusion increases exercise performance in SCD individuals [55]. Finally, the genotype (SS or SC) was also an independent predictor of the 6-MWT distance.

Then, we explored the determinants of the 6-MWT distance in each genotype. In SS patients, univariate analysis showed a positive correlation between the 6-MWT distance and both step frequency and Tmax, while hematocrit was nearly correlated with the 6-MWT distance (*p* = 0.06). In SC patients, both step frequency and hematocrit were positively correlated with the 6-MWT distance. However, in both genotypes, only step frequency remained an independent predictor of the 6-MWT distance. Considering the strong relationship between step frequency and walking speed [46,47,48], it is not surprising that step frequency remained an independent predictor of the 6-MWT distance. On the contrary, hematocrit did not remain an independent predictor of the 6-MWT when each genotype was considered separately. Our results contrast with those of Waltz et al. [20] and Marinho et al. [17], who found a positive relationship between the 6-MWT performance and the level of anemia in SS patients. These differences with our study could be explained by the difference in age (children vs. adults). Even though Tmax and hematocrit were not independent predictors of the 6-MWT distance in SS and SC separately in our study, both parameters should still be considered in the rehabilitation of SCD patients as they are closely related to the choice of step frequency. Indeed, anemia is a major factor in the reduction of exercise capacity in SS patients [53,54], and it should be a major target during exercise intervention. Moreover, skeletal muscle function is strongly implicated in step frequency and walking speed in healthy subjects [45,56] and a positive correlation between maximal isometric torque and functional capacity in chronic disease patients [27,28,29,30,31,32,57]. Therefore, similarly to anemia, skeletal muscle dysfunction should be considered as a therapeutic target in exercise rehabilitation in SCD patients as it was previously described that skeletal muscle function can be improved by endurance training [58,59].

## 5. Conclusions

In conclusion, our results showed that skeletal muscle dysfunction would be implicated in the reduced functional capacity of SCD patients, especially in SS, even though the 6-MWT did not result in skeletal muscle fatigue. Recently, an increasing number of studies showed that SCD patients display a profound skeletal muscle dysfunction that seems to contribute to the reduction of the functional capacity and quality of life of the patients. Further studies should be conducted to better characterize the relationship between skeletal muscle dysfunction and reduced functional capacity in SCD patients, in order to define intervention to improve muscle function. In addition, present results tend to indicate that SCD patients freely chose lower step frequency to minimize energy expenditure and minimize muscle fatigue during the 6-MWT.

## Figures and Tables

**Table 1:** Characteristics of subjects. BP: blood pressure. VOC: Vaso-occlusive crisis. ACS: Acute chest syndrome. *: *p* < 0.05 vs. AA; ***: *p* < 0.001 vs. AA; ^#^: *p* < 0.0001 vs. AA; ^$^: *p* < 0.0001 vs. AA and SC.

	AA	SS	SC
Men	10 (53%)	12 (50%)	6 (33%)
Women	9 (47%)	12 (50%)	12 (67%)
Age (years)	32 ± 9	27 ± 8	27 ± 12
Height (cm)	172 ± 10	172 ± 8	167 ± 8
Weight (kg)	73.1 ± 12.2	63.5 ± 9.1 *	64.1 ± 17.1
Heart rate (bpm)	74 ± 12	73 ± 8	77 ± 12
SpO_2_ (%)	97 ± 1	95 ± 3	95 ± 5
Systolic BP (mmHg)	125 ± 13	116 ± 16	120 ± 16
Diastolic BP (mmHg)	86 ± 9	75 ± 9 ***	78 ± 8 *
Hematocrit (%)	43.4 ± 4.4	26.2 ± 3.6 ^$^	32.6 ± 3.0 ^#^
Hemoglobin (g/dL)	14.3 ± 1.4	9.00 ± 1.2 ^$^	11.6 ± 1.1 ^#^
RBC (10^12^/L)	4.95 ± 0.6	2.95 ± 0.7 ^$^	4.44 ± 0.8
Leucocytes (10^9^/L)	5.39 ± 1.7	7.22 ± 2.3 *	6.55 ± 2.5
Hydroxyurea (n)		17	1 (6%)
VOC (n/5 years)		3.9 ± 3.0	2.5 ± 5.6
ACS (n/5 years)		0.7 ± 0.8	0
Physical activity (Met-min/sem)	2707 ± 3316	5282 ± 5815	1589 ± 1148

## Data Availability

The data that support the findings of the study are available on request from the corresponding author.

## References

[1] Ron T. (2012). Molecular Medicine.

[2] Weatherall D.J. (2010). The inherited diseases of hemoglobin are an emerging global health burden. Blood.

[3] Kato G.J., Piel F.B., Reid C.D., Gaston M.H., Ohene-Frempong K., Krishnamurti L., Smith W.R., Panepinto J.A., Weatherall D.J., Costa F.F. (2018). Sickle cell disease. Nat. Rev. Dis. Prim..

[4] Piel F.B., Howes R.E., Patil A.P., Nyangiri O.A., Gething P.W., Bhatt S., Williams T.N., Weatherall D.J., Hay S.I. (2013). The distribution of haemoglobin C and its prevalence in newborns in Africa. Sci. Rep..

[5] Hannemann A., Rees D.C., Tewari S.P., Gibson J.S. (2015). Cation Homeostasis in Red Cells from Patients With Sickle Cell Disease Heterologous for HbS and HbC (HbSC Genotype). EBioMedicine.

[6] Nagel R.L., Fabry M.E., Steinberg M. (2003). The paradox of hemoglobin SC disease. Blood Rev..

[7] Elmariah H., Garrett M.E., De Castro L.M., Jonassaint J.C., Ataga K.I., Eckman J.R., Ashley-Koch A., Telen M.J. (2014). Factors associated with survival in a contemporary adult sickle cell disease cohort. Am. J. Hematol..

[8] Lionnet F., Hammoudi N., Stojanovic K.S., Avellino V., Grateau G., Girot R., Haymann J.-P. (2012). Hemoglobin sickle cell disease complications: A clinical study of 179 cases. Haematologica.

[9] Machado R.F., Martyr S., Kato G.J., Barst R.J., Anthi A., Robinson M.R., Hunter L., Coles W., Nichols J., Hunter C. (2005). Sildenafil therapy in patients with sickle cell disease and pulmonary hypertension. Br. J. Haematol..

[10] Anthi A., Machado R.F., Jison M.L., Taveira-DaSilva A.M., Rubin L.J., Hunter L., Hunter C.J., Coles W., Nichols J., Avila N.A. (2007). Hemodynamic and Functional Assessment of Patients with Sickle Cell Disease and Pulmonary Hypertension. Am. J. Respir. Crit. Care Med..

[11] Alameri H.F., Aleem A., Kardas W., Jehangir A., Owais M., Al-Momen A. (2008). Dyspnea, pulmonary function and exercise capacity in adult Saudi patients with sickle cell disease. Saudi Med. J..

[12] Ohara D., Ruas G., Walsh I.A.P., Castro S.S., Jamami M. (2014). Lung function and six-minute walk test performance in individuals with sickle cell disease. Braz. J. Phys. Ther..

[13] Marouf R., Behbehani N., Zubaid M., Al Wazzan H., El Muzaini H., Abdulla R., Mojiminiyi O.A., Adekile A.D. (2014). Transthoracic Echocardiography and 6-Minute Walk Test in Kuwaiti Sickle Cell Disease Patients. Med. Princ. Pr..

[14] Melo H.N., Stoots S.J.-M., Pool M.A., Carvalho V.O., Almeida L.O.C., Aragão M.L.D.C., Agyemang C., Cipolotti R. (2017). Physical activity level and performance in the six-minute walk test of children and adolescents with sickle cell anemia. Rev. Bras. Hematol. Hemoter..

[15] Enright P.L. (2003). The Six-Minute Walk Test. Respir. Care.

[16] Connes P., Machado R., Hue O., Reid H. (2011). Exercise limitation, exercise testing and exercise recommendations in sickle cell anemia. Clin. Hemorheol. Microcirc..

[17] Marinho C.D.L., Maioli M.C.P., Soares A.R., Bedirian R., De Melo P.L., Guimarães F.S., Ferreira A.D.S., Lopes A.J. (2016). Predictive models of six-minute walking distance in adults with sickle cell anemia: Implications for rehabilitation. J. Bodyw. Mov. Ther..

[18] Brousse V., Pondarre C., Arnaud C., Kamden A., De Montalembert M., Boutonnat-Faucher B., Bourdeau H., Charlot K., Grévent D., Verlhac S. (2020). One-Fifth of Children with Sickle Cell Anemia Show Exercise-Induced Hemoglobin Desaturation: Rate of Perceived Exertion and Role of Blood Rheology. J. Clin. Med..

[19] Campbell A., Minniti C.P., Nouraie M., Arteta M., Rana S., Onyekwere O., Sable C., Ensing G., Dham N., Luchtman-Jones L. (2009). Prospective evaluation of haemoglobin oxygen saturation at rest and after exercise in paediatric sickle cell disease patients. Br. J. Haematol..

[20] Waltz X., Romana M., Hardy-Dessources M.-D., Lamarre Y., Divialle-Doumdo L., Petras M., Tarer V., Hierso R., Baltyde K.-C., Tressières B. (2013). Hematological and hemorheological Determinants of the Six-Minute Walk Test Performance in Children with Sickle Cell Anemia. PLoS ONE.

[21] Sachdev V., Kato G.J., Gibbs J.S.R., Barst R.J., Machado R.F., Nouraie M., Hassell K.L., Little J.A., Schraufnagel D.E., Krishnamurti L. (2011). Echocardiographic Markers of Elevated Pulmonary Pressure and Left Ventricular Diastolic Dysfunction Are Associated with Exercise Intolerance in Adults and Adolescents With Homozygous Sickle Cell Anemia in the US and UK. Circulation.

[22] Barst R.J., Mubarak K.K., Machado R.F., Ataga K.I., Benza R.L., Castro O., Naeije R., Sood N., Swerdlow P.S., Hildesheim M. (2010). Exercise capacity and haemodynamics in patients with sickle cell disease with pulmonary hypertension treated with bosentan: Results of the ASSET studies. Br. J. Haematol..

[23] Gordeuk V.R., Minniti C.P., Nouraie M., Campbell A., Rana S.R., Luchtman-Jones L., Sable C., Dham N., Ensing G., Prchal J.T. (2010). Elevated tricuspid regurgitation velocity and decline in exercise capacity over 22 months of follow up in children and adolescents with sickle cell anemia. Haematologica.

[24] Ravelojaona M., Féasson L., Oyono-Enguéllé S., Vincent L., Djoubairou B., Essoue C.E., Messonnier L.A. (2015). Evidence for a Profound Remodeling of Skeletal Muscle and Its Microvasculature in Sickle Cell Anemia. Am. J. Pathol..

[25] Waltz X., Pichon A., Lemonne N., Mougenel D., Lalanne-Mistrih M.-L., Lamarre Y., Tarer V., Tressières B., Etienne-Julan M., Hardy-Dessources M.-D. (2012). Normal Muscle Oxygen Consumption and Fatigability in Sickle Cell Patients Despite Reduced Microvascular Oxygenation and Hemorheological Abnormalities. PLoS ONE.

[26] Charlot K., Antoine-Jonville S., Moeckesch B., Jumet S., Romana M., Waltz X., Divialle-Doumdo L., Hardy-Dessources M.-D., Petras M., Tressières B. (2017). Cerebral and muscle microvascular oxygenation in children with sickle cell disease: Influence of hematology, hemorheology and vasomotion. Blood Cells Mol. Dis..

[27] Eken M.M., Richards R., Beckerman H., van der Krogt M., Gerrits K., Rietberg M., de Groot V., Heine M. (2020). Quantifying muscle fatigue during walking in people with multiple sclerosis. Clin. Biomech..

[28] Rovedder P.M.E., Borba G.C., Anderle M., Flores J., Ziegler B., Barreto S.S.M., Dalcin P.D.T.R. (2019). Peripheral muscle strength is associated with lung function and functional capacity in patients with cystic fibrosis. Physiother. Res. Int..

[29] Vivodtzev I., Pépin J.-L., Vottero G., Mayer V., Porsin B., Lévy P., Wuyam B. (2006). Improvement in Quadriceps Strength and Dyspnea in Daily Tasks After 1 Month of Electrical Stimulation in Severely Deconditioned and Malnourished COPD. Chest.

[30] Hendrican M.C., McKelvie R.S., Smith T., McCartney N., Pogue J., Teo K.K., Yusuf S. (2000). Functional capacity in patients with congestive heart failure. J. Card. Fail..

[31] Nyberg A., Törnberg A., Wadell K. (2016). Correlation between Limb Muscle Endurance, Strength, and Functional Capacity in People with Chronic Obstructive Pulmonary Disease. Physiother. Can..

[32] Bachasson D., Wuyam B., Pepin J.-L., Tamisier R., Levy P., Verges S. (2013). Quadriceps and Respiratory Muscle Fatigue Following High-Intensity Cycling in COPD Patients. PLoS ONE.

[33] Melo H.N., Stoots S.J.-M., Pool M.A., Carvalho V.O., Aragão M.L.D.C., Gurgel R.Q., Agyemang C., Cipolotti R. (2018). Objectively measured physical activity levels and sedentary time in children and adolescents with sickle cell anemia. PLoS ONE.

[34] Pinto D.M.R., Sacramento M.D.S.D., Santos P.H.S., Silva W.S., de Oliveira E.C., Gardenghi G., Ladeia A.M.T., Petto J. (2020). Physical exercise in sickle cell anemia: A systematic review. Hematol. Transfus. Cell Ther..

[35] Merlet A.N., Chatel B., Hourdé C., Ravelojaona M., Bendahan D., Féasson L., Messonnier L.A. (2019). How Sickle Cell Disease Impairs Skeletal Muscle Function: Implications in Daily Life. Med. Sci. Sports Exerc..

[36] Cleland C.L., Hunter R.F., Kee F., Cupples M.E., Sallis J.F., Tully M.A. (2014). Validity of the Global Physical Activity Questionnaire (GPAQ) in assessing levels and change in moderate-vigorous physical activity and sedentary behaviour. BMC Public Health.

[37] Petersen N.T., Taylor J., Butler J., Gandevia S.C. (2003). Depression of Activity in the Corticospinal Pathway during Human Motor Behavior after Strong Voluntary Contractions. J. Neurosci..

[38] ATS (2002). Committee on Proficiency Standards for Clinical Pulmonary Function Laboratories ATS Statement: Guidelines for the six-minute walk test. Am. J. Respir. Crit. Care Med..

[39] Borg E., Borg G., Larsson K., Letzter M., Sundblad B.-M. (2010). An index for breathlessness and leg fatigue. Scand. J. Med. Sci. Sports.

[40] Moheeb H., Wali Y.A., El-Sayed M.S. (2007). Physical fitness indices and anthropometrics profiles in schoolchildren with sickle cell trait/disease. Am. J. Hematol..

[41] Burr J.F., Bredin S.S.D., Faktor M.D., Warburton D.E.R. (2011). The 6-Minute Walk Test as a Predictor of Objectively Measured Aerobic Fitness in Healthy Working-Aged Adults. Physician Sportsmed..

[42] Geiger R., Strasak A., Treml B., Gasser K., Kleinsasser A., Fischer V., Geiger H., Loeckinger A., Stein J.I. (2007). Six-Minute Walk Test in Children and Adolescents. J. Pediatr..

[43] Hermens H.J., Freriks B., Disselhorst-Klug C., Rau G. (2000). Development of recommendations for SEMG sensors and sensor placement procedures. J. Electromyogr. Kinesiol..

[44] Huijben B., van Schooten K., van Dieën J., Pijnappels M. (2018). The effect of walking speed on quality of gait in older adults. Gait Posture.

[45] Lim Y.P., Lin Y.-C., Pandy M.G. (2017). Effects of step length and step frequency on lower-limb muscle function in human gait. J. Biomech..

[46] Pagliara R., Snaterse M., Donelan J.M. (2014). Fast and slow processes underlie the selection of both step frequency and walking speed. J. Exp. Biol..

[47] Hovington C.L., Nadeau S., Leroux A. (2009). Comparison of Walking Parameters and Cardiorespiratory Changes during the 6-Minute Walk Test in Healthy Sexagenarians and Septuagenarians. Gerontology.

[48] Yandell M.B., Zelik K.E. (2016). Preferred Barefoot Step Frequency is Influenced by Factors Beyond Minimizing Metabolic Rate. Sci. Rep..

[49] Ms J.A.S., Simonsick E.M., Ferrucci L. (2010). The Energetic Pathway to Mobility Loss: An Emerging New Framework for Longitudinal Studies on Aging. J. Am. Geriatr. Soc..

[50] Alexander N.B., Taffet G.E., Horne F.M., Eldadah B.A., Ferrucci L., Nayfield S., Studenski S. (2010). Bedside-to-Bench conference: Research agenda for idiopathic fatigue and aging. J. Am. Geriatr. Soc..

[51] Twomey R., Aboodarda S.J., Krüger R.L., Culos-Reed S.N., Temesi J., Millet G.Y. (2017). Neuromuscular fatigue during exercise: Methodological considerations, etiology and potential role in chronic fatigue. Neurophysiol. Clin. Neurophysiol..

[52] Barbosa J., Bruno S., Cruz N., De Oliveira J., Ruaro J., Guerra R. (2016). Perceived fatigability and metabolic and energetic responses to 6-minute walk test in older women. Physiotherapy.

[53] van Beers E.J., van der Plas M.N., Nur E., Bogaard H.-J., van Steenwijk R.P., Biemond B.J., Bresser P. (2014). Exercise tolerance, lung function abnormalities, anemia, and cardiothoracic ratio in sickle cell patients. Am. J. Hematol..

[54] Liem R.I., Nevin M.A., Prestridge A., Young L.T., Thompson A.A. (2009). Functional capacity in children and young adults with sickle cell disease undergoing evaluation for cardiopulmonary disease. Am. J. Hematol..

[55] Charache S., Bleecker E.R., Bross D.S. (1983). Effects of blood transfusion on exercise capacity in patients with sickle-cell anemia. Am. J. Med..

[56] Hoff J., Tjønna A.E., Steinshamn S., Høydal M., Richardson R.S., Helgerud J. (2007). Maximal Strength Training of the Legs in COPD. Med. Sci. Sports Exerc..

[57] Bonnevie T., Allingham M., Prieur G., Combret Y., Debeaumont D., Patout M., Cuvelier A., Viacroze C., Muir J.-F., Medrinal C. (2019). The six-minute stepper test is related to muscle strength but cannot substitute for the one repetition maximum to prescribe strength training in patients with COPD. Int. J. Chronic Obstr. Pulm. Dis..

[58] Merlet A.N., Messonnier L.A., Coudy-Gandilhon C., Bechet D., Gellen B., Rupp T., Galacteros F., Bartolucci P., Féasson L. (2019). Beneficial effects of endurance exercise training on skeletal muscle microvasculature in sickle cell disease patients. Blood.

[59] Merlet A.N., Féasson L., Bartolucci P., Hourdé C., Schwalm C., Gellen B., Galactéros F., Deldicque L., Francaux M., Messonnier L.A. (2020). Muscle structural, energetic and functional benefits of endurance exercise training in sickle cell disease. Am. J. Hematol..

